# Internal Modifications to Optimize Pollution and Emissions of Internal Combustion Engines through Multiple-Criteria Decision-Making and Artificial Neural Networks

**DOI:** 10.3390/ijerph182312823

**Published:** 2021-12-05

**Authors:** María Isabel Lamas Galdo, Javier Telmo Miranda, José Manuel Rebollido Lorenzo, Claudio Giovanni Caccia

**Affiliations:** 1 Escuela Politécnica de Ingeniería de Ferrol, Universidade da Coruña, 15403 Ferrol, Spain; 2 Escuela Técnica Superior de Ingenieros Industriales, UNED, 28040 Madrid, Spain; jtelmo@ieec.uned.es; 3 IES de Valga, 36645 Valga, Spain; navalrebollido@hotmail.com; 4 Department of Aerospace Engineering, Politecnico di Milano, 20156 Milan, Italy; claudiogiovanni.caccia@polimi.it

**Keywords:** engine, emissions, consumption, artificial neural network, multi-criteria decision-making, computational fluid dynamics

## Abstract

The present work proposes several modifications to optimize both emissions and consumption in a commercial marine diesel engine. A numerical model was carried out to characterize the emissions and consumption of the engine under several performance parameters. Particularly, five internal modifications were analyzed: water addition; exhaust gas recirculation; and modification of the intake valve closing, overlap timing, and cooling water temperature. It was found that the result on the emissions and consumption presents conflicting criteria, and thus, a multiple-criteria decision-making model was carried out to characterize the most appropriate parameters. In order to analyze a high number of possibilities in a reasonable time, an artificial neural network was developed.

## 1. Introduction

Though compression ignition engines are widely used in marine propulsion due to their important advantages, especially efficiency, their pollutant emissions threaten public health. Particularly, NO_x_ and SO_x_ are considered especially damaging [1,2,3,4]. In the marine field, even stricter limitations imposed by the IMO (International Maritime Organization) and other organizations regulate emissions from ships [5,6,7,8]. Regarding SO_x_, the maximum content in the fuels is limited for ships that do not have any post-treatment device. Regarding NO_x_, IMO establishes even stricter maximum emissions depending on the engine and region. According to this, many NO_x_ reduction measures have been developed along the years. Briefly, these measures can be classified into primary and secondary. Primary measures focus on avoiding NO_x_ formation during combustion, whereas secondary measures remove NO_x_ from the flue gases. Between the most employed primary measures, one can refer to modifications in the injection system, exhaust gas recirculation (EGR), water addition, modification of the distribution diagram, modification of the working pressures, and other parameters. An important handicap is that most of these measures reduce NO_x_ emissions at the expense of incrementing specific fuel consumption (SFC) and/or other emissions. In this regard, MCDM (multi-criteria decision-making) constitutes a formal tool for choosing between several alternatives which involve conflicting criteria. MCDM makes decision-making more effective when there are conflicting criteria, and minimizes human intervention in the decision process [9,10].

A decision-making process can be complemented by artificial neural networks (ANNs). ANNs are powerful data models that simulate the human brain, since these can learn the problem based on the available data, and develop a prediction. ANN models learn the structure of the process, and establish a relationship between the inputs and outputs. Recent works have indicated that the application of ANN techniques in decision-making problems is useful, and some researchers have used ANN models for MCDM analyses [11,12,13,14,15]. These prediction studies focused on ANN used together with MCDM, and applied this methodology to different aspects, such as supplier selection for industries [16,17,18,19], failure estimation in hydroelectric power plants [20], machine selection [21], store location selection [22], manufacturing technology investments [23], diagnosis of prostate cancer [24], etc. In these works, MCDM and ANN were used in an integrated manner, since MCDM is formulated, and ANNs are employed to learn the relation among alternatives and criteria, and rank the alternatives.

The present paper proposes a hybrid MCDM-ANN methodology to analyze several internal modifications to optimize both emissions and consumption from a compression ignition marine engine. The results obtained show that this methodology constitutes a robust tool for modeling the decision-making problem, since the decision-makers can analyze the different possibilities by changing the inputs, and observing the results in a fast and simple way. This model was applied to select the most appropriate water to fuel ratio, EGR rate, overlap timing, intake valves closings, and cooling water temperature.

## 2. Materials and Methods

This section firstly describes the engine analyzed, and the corresponding CFD analysis employed to obtain the data samples necessary to train, learn, and test the ANN. After that, the MCDM and ANN methodologies are treated.

### 2.1. Engine Analyzed and CFD Analysis

The present work analyzes the commercial diesel engine Wärtsilä 6L 46. This is a four-stroke engine with six in-line cylinders, and each cylinder has two inlet and two exhaust valves. The CFD analysis and validation with experimental results was developed and described in previous works [25,26,27,28,29,30], and thus, the procedure is not shown here in deep detail. The simulations were realized using the open software OpenFOAM. Turbulence was treated through the k-ε model. The fuel heat-up and evaporation were treated through the Dukowicz [31] model, and the fuel droplet breakup through the Kelvin–Helmoltz and Rayleigh–Taylor [32] models. As combustion, NO_x_ formation, and NO_x_ reduction, Ra and Reitz’s [33], Yang et al.’s [34], and Miller and Glarborg’s [35] kinetic schemes were employed, respectively.

**Figure 1:**
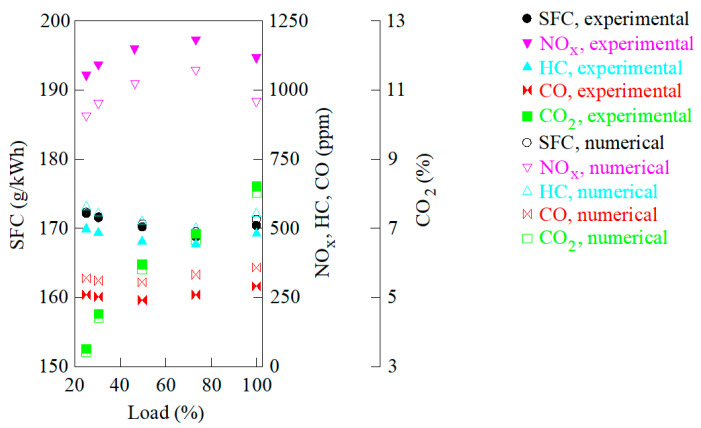
SFC and emissions.

**Figure 2:**
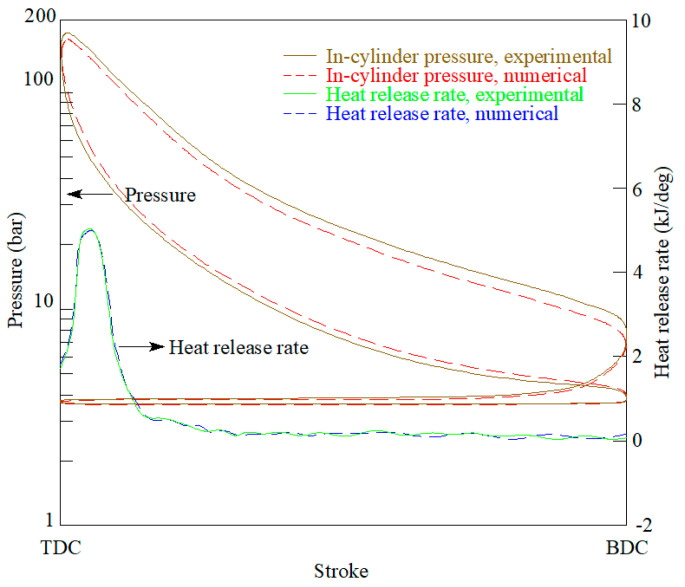
In-cylinder pressure and heat release rate at 100% load.

A comparison between the numerical and experimental results is illustrated in Figure 1 and Figure 2. Particularly, Figure 1 illustrates the emissions of NO_x_, carbon monoxide (CO), and hydrocarbons (HC), as well as SFC obtained numerically and experimentally at several loads, and Figure 2 illustrates the in-cylinder pressure and heat release rate obtained numerically and experimentally at 100% load. Other loads provided similar results, and thus, are not shown again. Both figures provide a satisfactory correspondence between the experimental results and the numerical ones provided by the CFD model.

**Figure 3:**
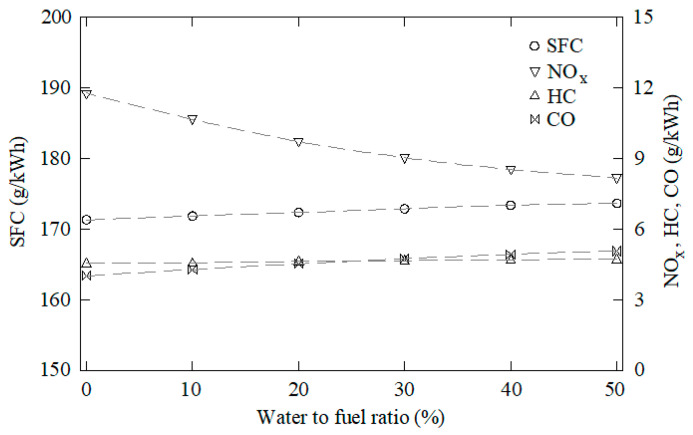
SFC, NO_x_, CO, and HC emissions against the water to fuel ratio. EGR rate = 0, overlap timing = 96°, intake valves closing = 568°, cooling water temperature = 76 °C.

**Figure 4:**
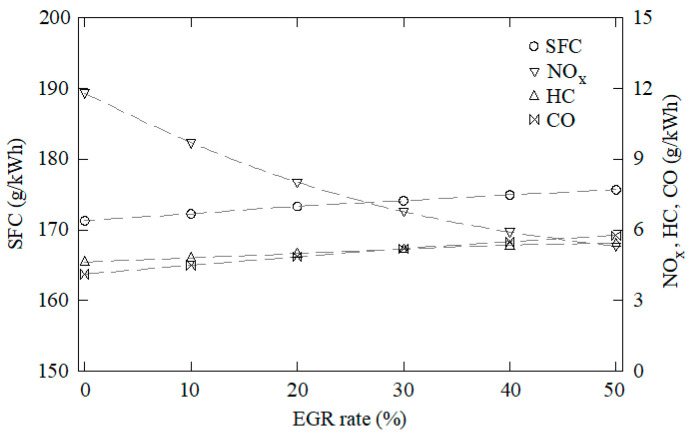
SFC, NO_x_, CO, and HC emissions against the EGR rate. Water to fuel ratio = 0, overlap timing = 96°, intake valves closing = 568°, cooling water temperature = 76 °C.

**Figure 5:**
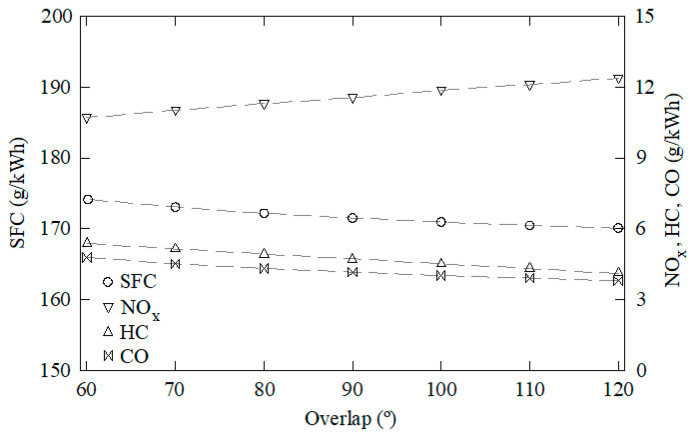
SFC, NO_x_, CO, and HC emissions against the overlap timing. EGR rate = 0, water to fuel ratio = 0, intake valves closing = 568°, cooling water temperature = 76 °C.

**Figure 6:**
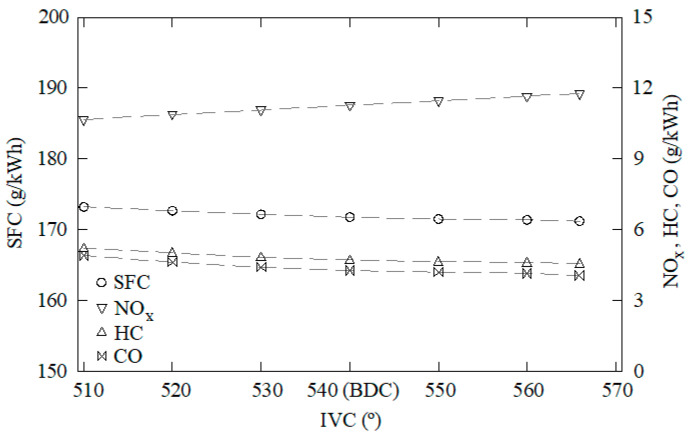
SFC, NO_x_, CO, and HC emissions against the intake valves closing instant. EGR rate = 0, water to fuel ratio = 0, overlap timing = 96°, cooling water temperature = 76 °C.

**Figure 7:**
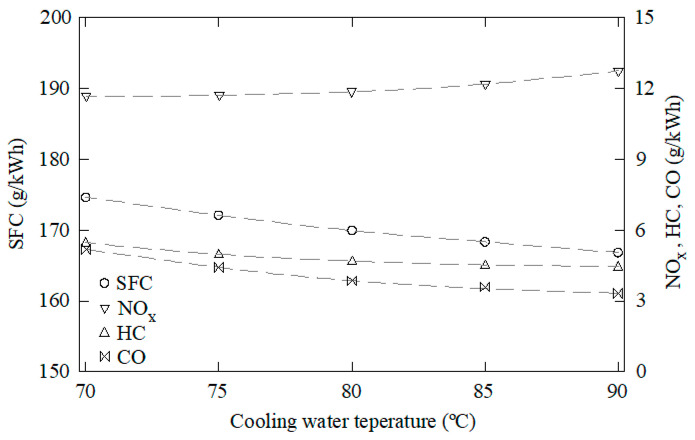
SFC, NO_x_, CO, and HC emissions against the cooling water temperature. EGR rate = 0, water to fuel ratio = 0, overlap timing = 96°, intake valves closing = 568°.

The data obtained through this CFD model were used as samples to train, validate, and test the ANN. One-hundred and forty-five cases were characterized through CFD using water to fuel ratios between 0 and 50%, EGR rates from 0 to 50%, overlap timings from 60 to 120°, intake valve closings from 510 to 570°, and cooling water temperatures from 70 to 90 °C. Some of these results are shown in Figure 3, Figure 4, Figure 5, Figure 6 and Figure 7. In these figures, emissions are represented in g/kWh instead ppm or %. The reason for this is to work with the same units as specific consumption. This way, all data that will be further employed in the MCDM model are introduced in the same unit. As can be seen in Figure 3, Figure 4, Figure 5, Figure 6 and Figure 7, NO_x_ emissions are reduced with increments of the water to fuel ratio and EGR rate, whereas NO_x_ emissions are incremented with decrements of the overlap, intake valves closing, and cooling water temperature. It is well known that NO_x_ is formed mainly due to the high temperatures reached along the combustion process [36,37]. If these temperatures are reduced, NO_x_ emissions are reduced too. Unfortunately, low combustion temperatures lead to lower power and, thus, higher consumption. Besides, lower combustion temperatures promote incomplete combustion, which is the main source of CO and HC emissions. According to these results, it can be seen that SFC, NO_x_, CO, and HC emissions constitute conflicting criteria, since none of the measures proposed in the present work are able to reduce all of them simultaneously.

### 2.2. MCDM Analysis

Taking into account the 145 alternatives analyzed through the CFD model, and the four criteria considered (SFC, NO_x_, CO, and HC emissions), a 145 × 4 data matrix can be constituted with 145 rows and 4 columns. This is shown in red in Table 1, in which the first and last rows are illustrated. This table also shows the water to fuel ratio (W), EGR rate (E), overlap timing (O), intake valves closing (I), and cooling water temperature (C) corresponding to each alternative.

An important issue in MCDM methods is establishing the criteria weights. The criteria weights represent the degree of importance of each criterion. Although some objective methods are available in the literature, subjective methods are recommended to establish the criteria weights, since these are directly defined by experts in the field [10,38], *i.e*., the experts have an important participation, and the weights are assigned based on experience and knowledge. On the contrary, objective methods are based in mathematical expressions that do not involve expertise or the experience of experts. In the present work, two main requirements were considered, consumption and emissions. A 20% importance was provided to consumption, and 80% to emissions. Regarding emissions, the importance of NO_x_, CO, and HC was distributed equally, *i.e*., 33.3% for each one. To summarize, these values in per-unit basis are shown in Table 2. Logically, each column in Table 1 sums 1 for the requirements. Regarding sub-requirements, the value of the part of the column corresponding to SFC is 1, and the part of the column corresponding to emissions sums 1 too. The weight of each criterion is obtained by multiplying the weight of the requirement by the weight of the sub-requirement, leading to 0.2, 0.267, 0.267, and 0.267 for SFC, NO_x_, CO, and HC emissions, respectively. Logically, these weights also sum 1. A sensibility analysis of the values assigned to the criteria weights will be shown in the results section.

Another important step is normalizing the decision matrix. Normalization is used to eliminate the units of each criterion so that all the criteria become dimensionless, and to set the ratings of different alternatives into the same range. Normalization changes the different measurable values into comparable similar ones. Many normalization techniques are available in the literature [39]. In the present work, the so-called linear max-min normalization technique was employed, according to which each normalized value, *V_ij_*, is given by:

(1) $$V_{ij}=1-\frac{X_{ij}}{X_{j,\max}^{}}$$

where by *X_ij_* is each value of the decision matrix. In the present work, the adequacy index was computed by the WSM (weighted sum method), according to which, the adequacy index is given by Equation (2). This procedure is also called SAW (simple additive weighting) or WLC (weighted linear combination). Taking into account the normalization procedure applied, the most appropriate alternative is the one corresponding to the maximum *AI*.

(2) $$AI_{i}=\sum_{j=1}^{n}w_{j}V_{ij}$$

whereby *AI* is the adequacy index, *w_j_* is the weight of the *j*-th criterion, and *n* is the number of criteria.

### 2.3. ANN Analysis

In order to analyze a number of alternatives much higher than 145, an ANN was established using the software Matlab 2021b. The general structure of an ANN is based on interconnected nodes organized into three parallel layers: input; hidden; and output. Input nodes contain the independent variables, whereas output nodes contain the dependent ones. The ANN obtains information by the network through a learning process, similarly to the human brain, i.e., imitates the learning ability of the human brain. The network used in the present work is illustrated in Figure 4. Five independent variables were employed, the water to fuel ratio (W), EGR rate (E), overlap timing (O), intake valves closings (I), and cooling water temperature (C); and one output variable, the adequacy index (AI). The 145 cases analyzed through CFD were employed as samples to train, validate, and test the network.

There is no exact rule to define the number of hidden layers and hidden nodes. Several methods to decide the number of the hidden layers can be found in the literature, and the general recommendation is to employ only one hidden layer for most problems [16,40], and the multi-layered structure is only recommended for complex problems [20]. Regarding the number of neurons in the hidden layer, a low quantity of hidden layer neurons increases the error. On the other hand, a high quantity of hidden layer neurons may lead to high computational cost and over-fitting. In the present work, several networks with neurons between 3 and 25 were tested to determine the optimum number of hidden layers for the present work. It was found that the most accurate results were obtained with 16 neurons.

**Figure 8:**
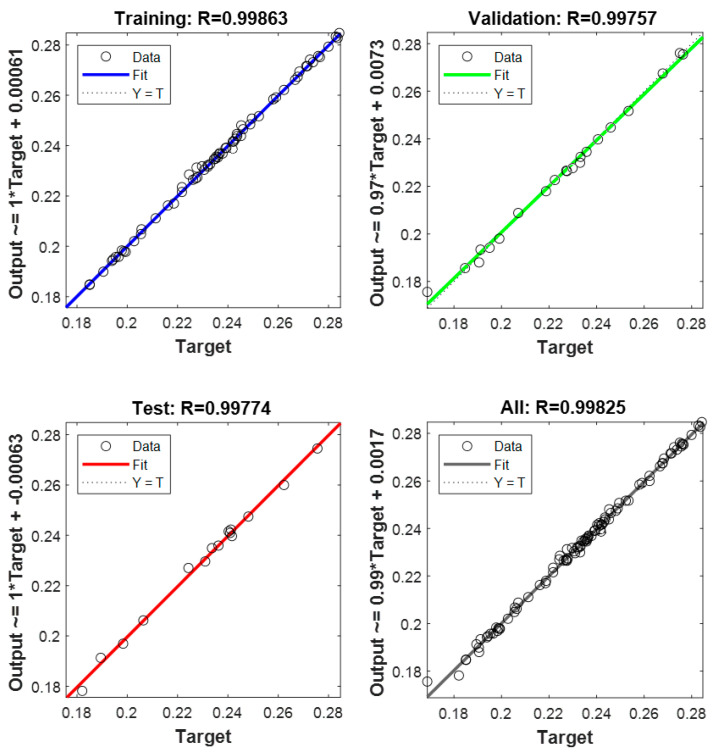
Regression graphs of the ANN.

As mentioned above, the 145 alternatives analyzed through the CFD model were employed to train, validate, and test the ANN. 101 samples, randomly selected, were used to train the network, 22 to validate it, and 22 to test it. The performance of the network is summarized in Figure 8. This figure illustrates the training, validation, and test regression plots. As can be seen, the curves of the four images are basically diagonal, thus, providing a good data fitting. The global R value, 0.99825, indicates an appropriate fit, since the optimum is R = 1, showing that these results are suitable for the operating conditions analyzed.

## 3. Results

**Figure 9:**
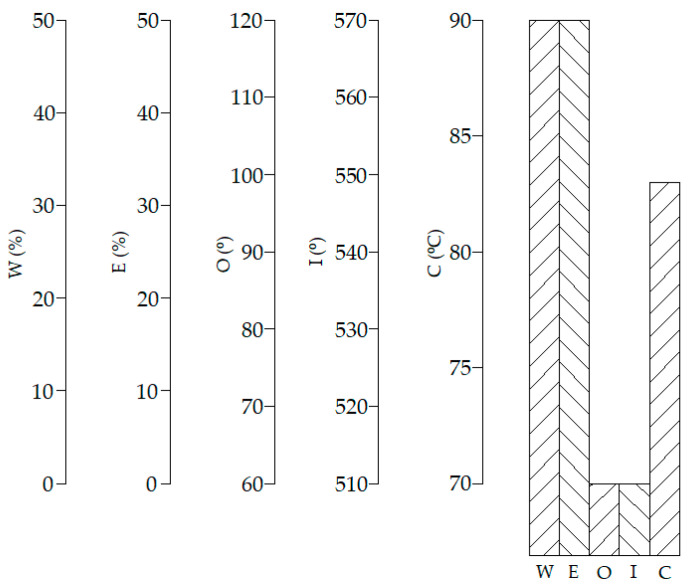
Most appropriate option provided by the hybrid MCDM-ANN model.

Using this hybrid MCDM-ANN model, 203,244,741 alternatives were analyzed under water to fuel ratios between 0 and 50%, EGR rates from 0 to 50%, overlap timings from 60 to 120°, intake valve closings from 510 to 570°, and cooling water temperatures from 70 to 90 °C. The advantage of employing ANNs is that it is possible to analyze this huge quantity of alternatives with a low computational cost. On the other hand, analyzing 203,244,741 cases using CFD is too computationally expensive for the current technology. Figure 9 shows the most appropriate option provided, corresponding to the maximum *AI* of these alternatives analyzed. The most appropriate configuration illustrated in this figure presents 50% water addition, 50% EGR rate, 60° overlap timing, 510° intake valve closing, and 78°C cooling water temperature. This solution was obtained using the criteria weights shown in Table 2. It is useful to realize a sensitivity analysis of these criteria weights. According to this, Table 3 shows the most appropriate configuration under different weights assigned to the consumption. In this sensitivity analysis, the emissions were assigned equally with the remaining weight. As can be seen, if the SFC weight is increased, the water to fuel ratio and EGR rate decrease. The reason for this is the effect of these parameters on combustion, which is incremented. The NO_x_ variation with water addition and EGR rate are important too, especially with the EGR rate. The values of the overlap timing, intake valves closing, and cooling water temperature are a compromise between emissions and consumption. The scavenging of the combustion gases is related to the overlap period. Short overlap periods lead to excessive quantities of residual gas in the cylinder, thus, reducing NO_x_ emissions, but increasing HC and CO due to a poorer combustion. Regarding the intake valves closing, if these valves are closed early, less air enters the cylinder. This leads to low combustion temperatures and, thus, NO_x_ emissions. On the contrary, CO and HC emissions are high at early intake valve closings. Regarding the cooling water temperature, this highly affects the temperature inside the cylinder. According to this, low values of the cooling water temperature lead to low NO_x_ emissions, but high CO and HC emissions, as well as consumption.

## 4. Conclusions

This work proposes a hybrid MCDM-ANN model for selecting the most appropriate working parameters in the commercial marine engine Wärtsilä 6L 46. The aim is to reduce emissions and consumption as much as possible, and five parameters were analyzed: water addition; exhaust gas recirculation; and modification of the intake valve closing, overlap timing, and cooling water temperature. Since it is impossible to reduce both emissions and consumption simultaneously modifying these parameters, an MCDM model was developed to select the most appropriate option. Also, in order to analyze a high quantity of alternatives, an ANN was developed. 145 cases were employed to train, validate, and test the ANN, and the data corresponding to these 145 cases were obtained through a validated CFD model. A satisfactory prediction accuracy was obtained for the ANN. The main advantage of the CFD model is that it provides data avoiding expensive and laborious experimental setups. In order to obtain these data experimentally, it would be necessary to modify the engine for each alternative analyzed. The present work shows the utility of ANNs to analyze engines, and provide a useful tool for decision-making when selecting engine parameters.

In future works, this methodology will be employed to analyze other important parameters, such as injection timing, injection pressure, intake pressure, etc. Five relevant measures were chosen for the present work, but the model can be extended to include as many measures as data are possible to obtain.

## Figures and Tables

**Table 1:** Decision matrix.

Case (*i*)	W (%)	E (%)	O (°CA)	I (°CA)	C (°CA)	Criterion (*j*)			
						*j* = 1 SFC	*j* = 2 NO_x_	*j* = 3 CO	*j* = 4 HC
1	0	0	60	510	70	**179.34**	**9.75**	**6.51**	**6.71**
2	10	0	60	510	70	**179.88**	**8.65**	**6.77**	**6.72**
.	.	.	.	.	.	**.**	**.**	**.**	**.**
.	.	.	.	.	.	.	**.**	**.**	**.**
100	50	50	120	570	90	**172.17**	**3.44**	**5.60**	**4.87**

**Table 2:** Criteria weights, per unit basis.

Requirement (*α*)	Sub-Requirement (*β*)
SFC (0.2)	SFC (1)
Emissions (0.8)	NO_x_ (0.333)
	CO (0.333)
	HC (0.333)

**Table 3:** Most appropriate option under several criteria weights for the consumption.

A_SFC_	W (%)	E (%)	O (°)	I (°)	C (°C)
20	50	50	60	510	86
30	46	50	104	548	90
40	32	39	120	570	90
50	22	28	120	570	90
